# An Updated Review of BiCuSeO-Based Thermoelectric Materials

**DOI:** 10.3390/mi16060703

**Published:** 2025-06-12

**Authors:** Haitao Zhang, Bo Feng, Suoluosu Yang, Ruolin Ruan, Rong Zhang, Tongqiang Xiong, Biyu Xu, Zhipeng Zheng, Guopeng Zhou, Yang Zhang, Kewei Wang, Yin Zhong, Yanhua Fan, Xiaoqiong Zuo

**Affiliations:** 1 Institute of Engineering and Technology, Hubei University of Science and Technology, Xianning 437100, China; 2 School of Mechanical and Electrical Engineering, Wuhan Donghu University, Wuhan 430070, China; 3 Hubei Xiangcheng Intelligent Electromechanical Research Institute Co., Ltd., Xianning 437100, China; 4 Hubei MAGNIFICENT New Material Technology Co., Ltd., Xiangyang 441200, China; 5 Jiangsu MAGNIFICENT New Material Technology Co., Ltd., Yancheng 224000, China; 6 School of Electronic and Electrical Engineering, Wuhan Textile University, Wuhan 430200, China

**Keywords:** BiCuSeO, thermoelectric material, layered structure, *ZT* value

## Abstract

Since 2010, BiCuSeO has emerged as a captivating subject of investigation within the realm of thermoelectric materials. Its allure lies in a remarkable confluence of characteristics: a distinctive natural super-lattice structure, an elevated Seebeck coefficient, and a low thermal conductivity, all of which have collectively piqued the intense interest of scientists worldwide. Over the subsequent eight-year period, an extensive array of research endeavors has been meticulously carried out, delving deep into the multifaceted properties of BiCuSeO and exploring avenues for performance enhancement. In this comprehensive review, we embark on a detailed exploration of the fundamental properties of BiCuSeO, encompassing its preparation methodologies, as well as its thermoelectric and mechanical attributes. A thorough synthesis of diverse strategies for optimizing the composition and structure of BiCuSeO is presented, elucidating how these modifications contribute to the enhancement of its thermoelectric and mechanical performance. Finally, the current state of research on N-type BiCuSeO is systematically summarized, offering a panoramic view of the advancements and challenges in this particular area.

## 1. Introduction

Thermoelectric materials can transform heat energy and electrical energy into one another. They have good application prospects in thermoelectric power generation by utilizing low-grade waste heat of high-temperature industries and dispersed distributed waste heat, and they have been a hot topic in the field of materials research for a long time [1,2]. Oxide thermoelectric materials are typically composed of abundant elements in the earth’s crust, free of heavy metals like lead, tellurium, and bismuth. This avoids potential hazards to humans and the environment, aligning with the concepts of green chemistry and sustainable development. Oxide materials exhibit high melting points and strong chemical bonds, maintaining structural stability in high-temperature and oxidizing environments. They are suitable for harsh scenarios like industrial waste heat recovery and automotive exhaust power generation, where metal-based materials are prone to oxidation and failure. Raw materials are inexpensive and easily accessible, and production processes are relatively simple, requiring no complex purification or vacuum environments. This significantly reduces preparation costs and facilitates large-scale industrial applications. Oxide materials exhibit good chemical compatibility with insulating substrates like ceramics and glass and can be integrated into complex devices through processes such as printing and coating, making them suitable for modular design in flexible electronics or micro-energy systems [3,4,5]. Among them, BiCuSeO-based thermoelectric materials are the most excellent oxygenated thermoelectric materials so far and are considered a new type of thermoelectric conversion material with one of the most promising application prospects [6,7,8].

BiCuSeO-based thermoelectric materials exhibit very low thermal conductivity (0.6 Wm^−1^K^−1^ at room temperature and 0.4 Wm^−1^K^−1^ at 923 K) due to their special natural super-lattice structure and low Young’s modulus [9,10,11]. The Seebeck coefficient is larger than 300 µVK^−1^ in the range of 300–923 K. At present, the main obstacle to improving the thermoelectric properties of BiCuSeO is its low electrical conductivity. In recent years, related research has mainly focused on how to improve the electrical conductivity of the system. Researchers improve the conductivity of the system by means of element doping [12,13,14], Cu vacancies [15], band gap regulation [16], and dual doping or texturing [17,18,19,20,21], and correspondingly improve the thermoelectric properties of BiCuSeO. At present, the value of *ZT* of BiCuSeO-based thermoelectric materials can reach 1.5, which is comparable to that of metal–semiconductor alloys, and attracts the wide attention of scholars [18].

In this review, we commence by introducing the fundamental properties of BiCuSeO, with a particular focus on its crystal structure. Subsequently, we delve into its preparation methodologies, encompassing high-temperature solid-state reaction synthesis, mechanical alloying, self-propagating synthesis, and high-pressure synthesis, detailing the processes from powder fabrication to bulk material formation. Following this, we summarize a plethora of composition adjustment techniques aimed at enhancing its thermoelectric and mechanical properties. These strategies include low-valence element doping, isovalent doping, vacancy engineering, and dual-site doping, among others. Next, we explore various structural modification approaches for improving the thermoelectric and mechanical performance of BiCuSeO, such as modulation doping, grain refinement, and composite material fabrication. Finally, we provide a comprehensive overview of the current research status of N-type BiCuSeO, highlighting the key findings and ongoing challenges in this field. Finally, the future research directions of BiCuSeO-based thermoelectric materials are summarized, including the ways to improve the thermoelectric properties of P-type BiCuSeO, the enlargement of the negative range of Seebeck coefficient for N-type BiCuSeO research, and the corresponding improvement of thermoelectric properties.

## 2. The Basic Properties of BiCuSeO

### 2.1. The Special Natural Super-Lattice Structure of BiCuSeO

**Figure 1:**
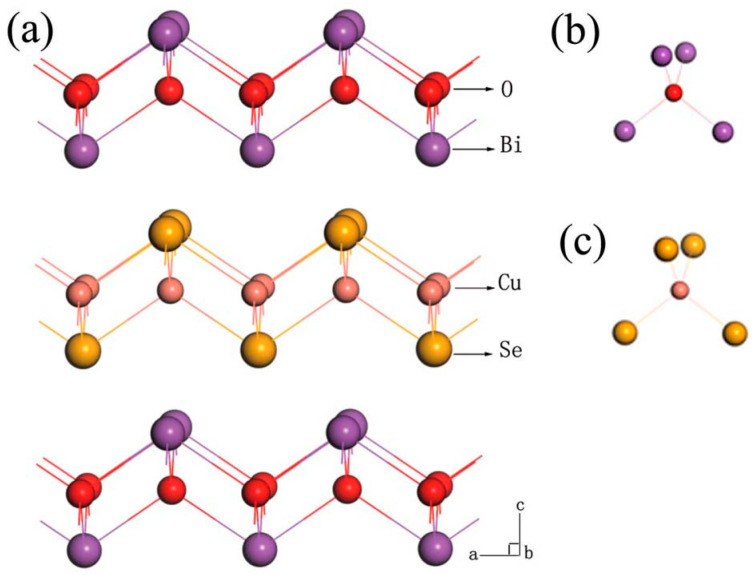
(**a**) Crastal Structure of BiCuSeO, (**b**) Bi_4_O tetrahedra, and (**c**) CuSe_4_ tetrahedra [16].

BiCuSeO has drawn significant attention in the field of thermoelectric materials due to its unique structure and excellent thermoelectric properties. The study of its structure–property relationship is crucial for optimizing its performance. The alternating stacking of the insulating layer and the conductive layer in BiCuSeO forms a special natural super-lattice structure, which plays a key role in determining its thermoelectric behavior. To understand the impact of this structure on the material’s properties, detailed calculations of the density of states (DOS) are essential. BiCuSeO crystallizes in a layered ZrCuSiAs structure, and the space group *P*4*/nmm*. It exhibits a special natural super-lattice structure stacked alternately with the (Bi_2_O_2_)^2+^ layer (the insulating layer, also known as the carrier storage layer) and the (Cu_2_Se_2_)^2−^ layer (the conductive layer, also known as the carrier transporting layer), as shown in Figure 1. The (Bi_2_O_2_)^2+^ layer and the (Cu_2_Se_2_)^2−^ layer consist of Bi_4_O tetrahedra and CuSe_4_ tetrahedra, respectively [19]. The alternating stacking structure between the insulating layer and the conductive layer has a great influence on the performance. On the one hand, the corresponding quantum confinement effect makes the Seebeck coefficient of material very large. Although the insulating layer does not participate in conduction, in our DOS (density of states) calculation of the Bi-site substitution experiment, the PDOS (partial density of states) of Bi is almost unchanged before and after doping, while the change in the PDOS of Cu/Se is relatively large; however, the insulating layer can play the role of storing carriers [13]. For example, through Bi vacancies or doping the Bi site with low-valence elements can enhance carrier concentration, thereby enhancing the thermoelectric properties of BiCuSeO. The doping of Bi sites is also the main method to adjust the thermoelectric properties of BiCuSeO at present, mainly because Bi exhibits + 3 valence in BiCuSeO. Although Cu is located in the conductive layer, it exhibits +1 valence and cannot introduce holes by low-valence elements doping.

### 2.2. The Vickers Hardness of BiCuSeO

In the field of thermoelectric materials, mechanical properties are critical determinants for both processing feasibility and service durability, yet they have long been underexplored in BiCuSeO research. To bridge this gap, our study systematically investigates the Vickers hardness of BiCuSeO, a key mechanical property reflecting material resistance to plastic deformation. Understanding how synthesis methods and compositional modifications affect hardness is essential for optimizing its practical applications, especially in scenarios requiring structural stability during fabrication or operation. Mechanical properties have a great influence on the processing and service life of thermoelectric materials. Previous studies have ignored the mechanical properties of BiCuSeO. We systematically studied the Vickers hardness of BiCuSeO. The Vickers hardness of the samples prepared by solid-state reaction is ~181.32 HV, while that of the samples prepared by mechanical alloying (8 h) is ~226.65 HV. The reason lies in that the samples prepared by mechanical alloying have smaller grain sizes and correspondingly higher Vickers hardness due to fine grain strengthening. With the prolongation of mechanical alloying time, the grain size decreases, and the corresponding Vickers hardness increases (~270.37 HV for 16 h). As a kind of ceramic material, its Vickers hardness is far larger than that of bismuth telluride (~65.37 HV). By doping, Vickers hardness increases further because of solution strengthening. For 8%Sb/Te-doped BiCuSeO, the Vickers hardness increases to as high as 330.02 HV [20].

## 3. The Preparation Methods of BiCuSeO

**Figure 2:**
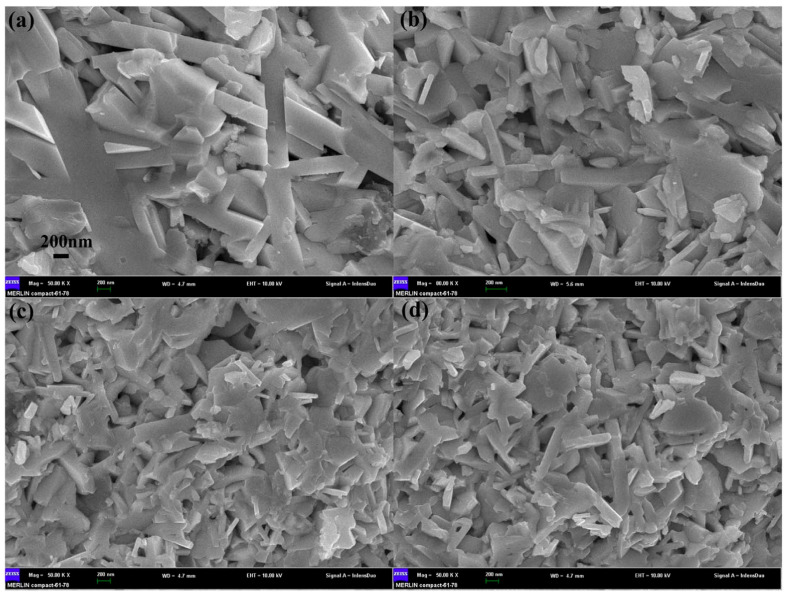
SEM of the fractured surfaces for Bi_0.92_Sb_0.08_CuSe_0.92_Te_0.08_O bulks ((**a**–**d**) stand for t = 5 h, 8 h, 12 h, 16 h, respectively) [17].

The synthesis methodology of BiCuSeO significantly influences its microstructure, phase purity, and ultimately its thermoelectric performance. Understanding the evolution of preparation techniques is crucial for optimizing material properties and advancing practical applications. This section systematically reviews the historical development and mechanistic insights of various synthesis methods for BiCuSeO, highlighting their advantages, limitations, and structural–property relationships. BiCuSeO was first synthesized through a solid-state reaction. The early synthesis time is long, and the density is not high. Later, a one- or two-step solid-state reaction synthesis was developed to obtain high-density samples [9,21]. The powder is first cold pressed into blocks, then annealed, then crushed and sintered into blocks. However, the process of this method is complicated, time-consuming, and energy-consuming. Mechanical alloying has become the main preparation method of BiCuSeO because of its simple process, time efficiency, and energy savings. We have also studied the grain size from different preparation processes. As shown in Figure 2, the average grain size of the annealed sample is 1481 nm, much larger than 952 nm for the BM sample with *t* = 8 h. With the prolongation of ball milling time, the average size of the grain significantly decreases, from 952 nm for *t* = 8 h to 598 nm for *t* = 12 h, and further down to 400 nm for 16 h [17]. When the particle size of the powder decreases, the grain size of the sintered product becomes smaller accordingly. Meanwhile, the increase in Cu vacancies and carrier concentration can enhance the electrical conductivity of BiCuSeO. This phenomenon can be attributed to the following mechanisms: smaller powder particles provide a larger specific surface area, which increases the driving force for atomic diffusion during sintering and promotes the formation of finer grains. Additionally, the reduction in particle size may introduce more defects (such as Cu vacancies) into the crystal structure, serving as additional charge carriers. Higher carrier concentration shortens the average distance for charge transport, thereby improving electrical conductivity. These effects collectively demonstrate the critical role of powder particle size control in optimizing the microstructural and electrical properties of BiCuSeO-based materials.

Self-propagating high-temperature synthesis (SHS) is also used to prepare BiCuSeO [22,23,24]. The SHS process involves the following steps: first, cold-pressing raw powders into bulk materials; then, controlling the temperature to trigger a self-propagating reaction. After the reaction is completed, the bulks are crushed, and the resulting powders are sintered. The advantage of SHS is that the self-propagating reaction process is very short (the reaction time takes only a few seconds), but it is not widely used because the process is not easy to control and needs to go through the process of crushing and pulverizing. In addition, flux synthesis is also used to synthesize BiCuSeO [25]. Put the powders in a crucible, cover the surface with excessive bismuth oxide powder, then heat it to 873 K for 10 h. After cooling to room temperature, the sample was taken out and bismuth oxide slag was separated. Then the block was crushed, and the powder was sintered. The advantage of this method is that BiCuSeO can be synthesized in air, but it consumes high energy and needs to go through bismuth oxide slag separation and bulk crushing, so it is seldom used. Z. Liu et al. have adopted high pressure to synthesize BiCuSeO and found that high pressure can reduce the band gap of BiCuSeO, thereby improving the carrier concentration and electrical performance [26]. The *PF* was increased from 21 µWm^−1^K^−1^ for 0.8 GPa to 80 µWm^−1^K^−1^ for 4 GPa at room temperature.

## 4. The Composition Adjustments of BiCuSeO

### 4.1. Low-Valence Element Doping for BiCuSeO

The optimization of thermoelectric properties in BiCuSeO heavily relies on rational doping strategies, which aim to tailor carrier concentration, adjust band structure, and manipulate phonon scattering. Given the material’s layered structure with distinct charge storage and transport layers, doping at the Bi site has emerged as a primary approach due to Bi’s trivalent state, which allows for effective carrier modulation via valence mismatch. This section systematically evaluates the doping mechanisms and performance outcomes of various elemental substitutions in BiCuSeO, emphasizing the structure–property relationships and key challenges in achieving high thermoelectric efficiency. Cu shows positive monovalence in BiCuSeO, Bi shows positive trivalence, Se and O show negative bivalence, while P-type doping (introduce holes) requires a lower valence of positive valence elements or a higher valence of negative valence elements. Yang et al. used positive tetravalent Sn-doped Bi sites, the maximum *ZT* (873 K) value was only 0.3 [27]. Luu et al. used positive divalent Cd and Zn to dope at the Cu site, and the thermoelectric properties were not improved [28]. At present, doping modification is mainly concentrated on the Bi site. Positive univalent or positive bivalent elements can be used for doping to improve carrier concentration and enhance electrical transport performance.

Alkaline earth metal elements (Mg [29], Ca [30,31], Sr [32], and Ba [33,34]) have been successfully used to dope BiCuSeO. Divalent M (Mg, Ca, Sr, Ba) can substitute the trivalent Bi site and increase carrier concentration. Among them, the doping effect of Ca, Sr, and Ba is comparatively close, which can increase the carrier concentration to 10^20^ cm^−3^ or even 10^21^ cm^−3^, while the effect of Mg doping is relatively weak, which can only increase the carrier concentration to 10^19^ cm^−3^. This is due to the relatively smaller atomic radius of Mg and the relatively low probability of orbital coincidence with other atoms after substituting the Bi site, resulting in relatively low doping efficiency. The doping effect of Ca and Sr is considerable, which can increase the *ZT* value to 0.90 (923 K) and 0.76 (873 K), respectively. The best is Ba doping, whose *ZT* value is as high as 1.1 (923 K), which is higher than that of all other elements. The important reason is that the atomic mass of Ba is larger. Compared with other elements, Ba atoms belong to “heavy elements”, which can scatter phonons more effectively, reduce the thermal conductivity, and improving electrical properties.

Alkali metals are also used to dope BiCuSeO. Na doping was studied by Zhang and Novitskii. Novitskii et al. [21] introduced Na with NaF as the raw material. Because of the negative valence of F, the effect of donor doping on BiCuSeO will be counteracted. The doping effect is not ideal. The highest *ZT* value is only ~0.42 (923 K), and the *ZT* value of some samples at high temperatures is even lower than that of the undoped samples. Zhang et al. [35] successfully introduced Na doping with Na_2_CO_3_ as raw material (the C element volatilizes in the form of CO_2_ in the preparation process). The carrier concentration of all doped samples (Na doping amount is x = 0.02, 0.04, 0.06, 0.08, 0.12, respectively) was 10^20^ cm^−3^, the power factor was increased to ~0.6 mWm^−1^K^−2^, and the maximum *ZT* value was 0.97 (873 K). Zhao et al. adopted a similar method to realize Na doping. The maximum *ZT* value increased to 0.91 (923 K) [36]. Lee et al. introduced K with K_2_O as raw material, but the doping efficiency was much lower than that of Na doping [37]. The highest carrier concentration was only 4.78 ×10^19^ cm^−3^, which may be due to the difference in radius between K and Bi ions. At room temperature, the electrical conductivity of the K-doped sample is obviously higher than that of the undoped sample, but with the increase in temperature, the conductivity decreases greatly, which makes the effect of K-doping unsatisfactory. Cs is used to dope with BiCuSeO using Cs_2_CO_3_ as raw material, and the maximum *ZT* value is 0.66 [21]. As a low-valence heavy element, Cs doping has not achieved good results, which may be due to the flux method used. If mechanical alloying is used to refine grain, it should be able to achieve better results.

Ag element has also been successfully used to dope BiCuSeO [38], but the increase in the electrical conductivity is only up to ~28 Scm^−1^, and the power factor is ~0.32 mWm^−1^K^−2^. Ag doping can obviously refine the grain size and reduce the lattice’s thermal conductivity. The highest *ZT* value can reach 0.68 (873 K). Some studies suggested that Ag should be doped at the Cu site instead of Bi [39]. The highest *ZT* value increased to 0.64. This should be a misconception, because the radius of the Ag ion (1.15 Å) is larger than that of Bi (1.03 Å), and the radius of the Bi ion is larger than that of Cu (0.77 Å), so Ag should be doped at the Bi site. The presence of CuSe/Cu_2_Se hybrids in this study also proves this. Pb doping can improve the electrical transporting performances [40,41,42]. A small amount of Pb doping can increase the carrier concentration to 10^20^ cm^−3^, the power factor is ~0.53 mWm^−1^K^−2^, and the *ZT* value can reach 0.95 (873 K). The power factor of Pb-doped samples is different from that of other elements-doped samples. The power factor of Pb-doped samples is relatively high in the whole temperature range (the power factor of other doped samples is generally very low in the low-temperature range, and increases sharply with the increase in temperature). In addition, the doping of the Pb element can obviously increase the grain size and improve the lattice thermal conductivity of the material. G. K. Ren et al. combined Pb doping with PbSe precipitates to improve the *ZT* to 1.3 at 873 K. Butt et al. attempted to dope with the Zn element [43]. The maximum power factor was up to ~0.35 mWm^−1^K^−2^ and the maximum *ZT* value was 0.65 (873 K). Farooq et al. doped the Bi site with the Cd element [44]; the highest *ZT* value of the 5% Cd-doped sample reached 0.98 (923 K), and the power factor reached ~0.60 mWm^−1^K^−2^ at 323 K and ~0.45 mWm^−1^K^−2^ at 923 K. Yb^2+^ was used to dope at the Bi site by Kang et al. [45]. The maximum power factor was up to ~0.28 mWm^−1^K^−2^, and the maximum *ZT* value was 0.62 (873 K).

Sayan Das et al. attempted to use a magnetic low-valence element (Mn^2+^) for doping because magnetic elements can increase the rotational entropy and increase the Seebeck coefficient; however, the improvement of thermoelectric properties is not obvious [46], with the highest being 0.4 at 773 K.

### 4.2. Equivalent Valence Element Doping for BiCuSeO

**Figure 3:**
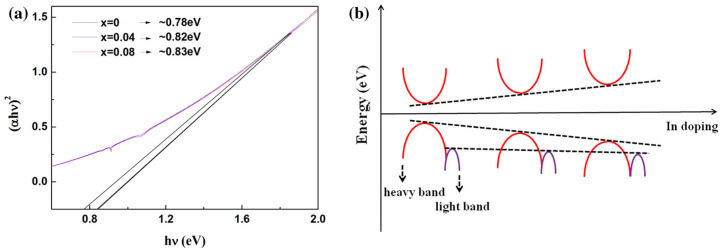
(**a**) The optical band gaps, and (**b**) the schematic diagram of proposed band model of BCSO samples [50].

**Figure 4:**
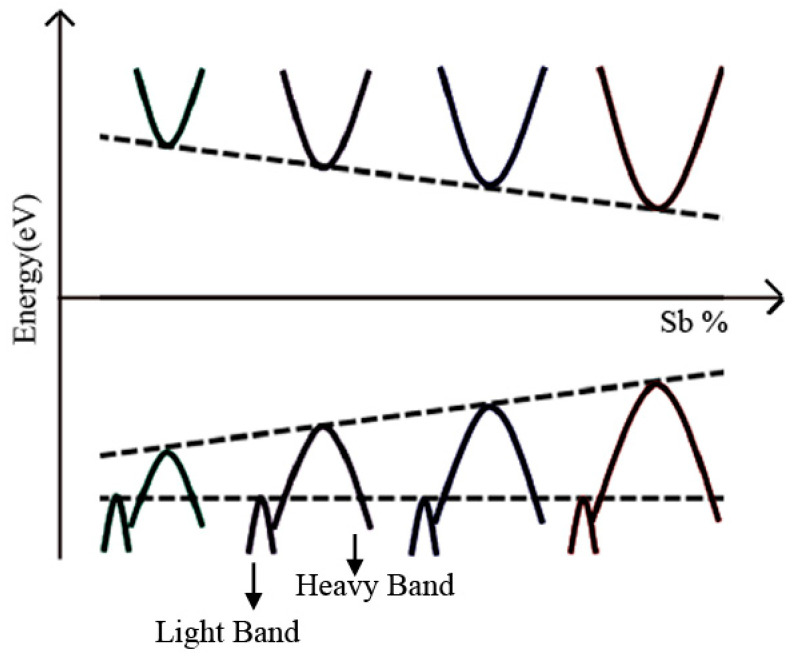
The proposed band model for the Bi_1-x_Sb_x_CuSeO samples [16].

**Figure 5:**
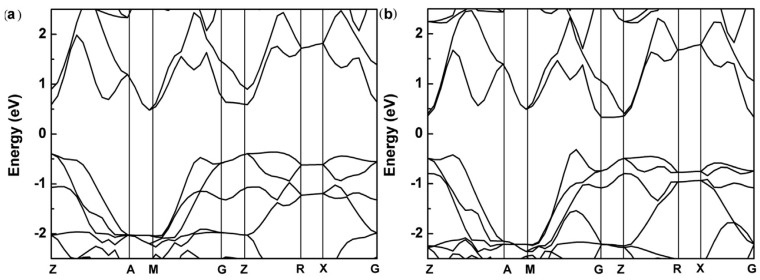
The calculated GGA-PBE band structure of BiCuSeO (**a**) and SbCuSeO (**b**) [16].

**Figure 6:**
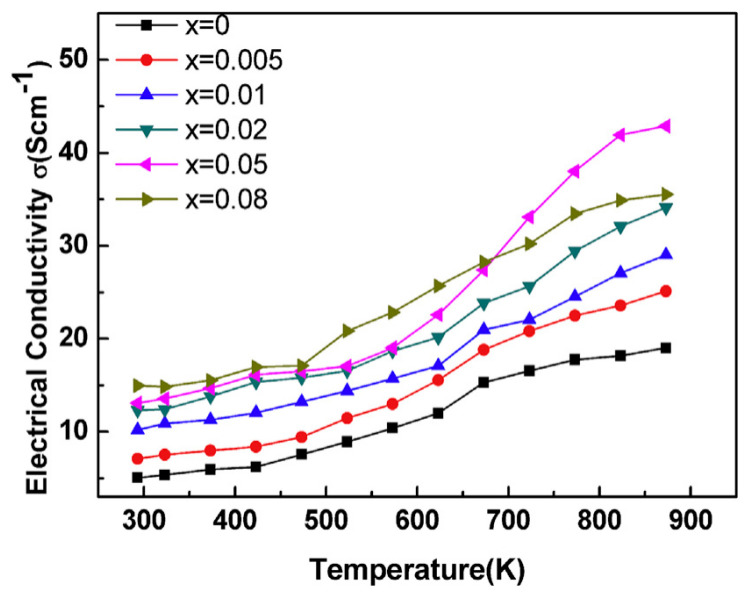
The Electrical conductivity of Sb-doped BiCuSeO [16].

The energy band of BiCuSeO is composed of light bands and heavy bands. The study of PbSe and PbTe shows that adjusting the structure of the mixed band can effectively improve electrical performance. The band gap of BiCuSeO is ~0.8 eV, and S, Se, and Te are congeners. Se can be substituted by S and Te. Although their valence states are the same, the difference in atomic weight will cause obvious changes in the energy band structure of BiCuSeO. When Te is used to dope Se, the band gap decreases to ~0.4 eV, and when S is used instead of Se, the band gap increases to ~1.10 eV [47,48]. The results of David et al. showed that when Te was doped at the Se site, the carrier concentration of BiCuSeO increased significantly, the electrical conductivity increased up to 40 S/cm, and the maximum *ZT* value reached 0.71 (923 K). The substitution of S at the Se site not only enlarges the band gap but also deteriorates the thermal stability of the material, making it difficult to obtain a single phase. La can also be used to dope BiCuSeO. La and Bi have the same valence (+3), and the band gap increases rapidly after La or In doping [49,50]. When La doping amount x = 0.125, the band gap has increased to ~1.3 eV, and the carrier concentration has decreased significantly. However, because the energy band of BiCuSeO is a mixture of light band and heavy band, the band gap becomes wider (as shown in Figure 3), the energy offset between heavy band and light band becomes smaller, and the contribution of light band increases, while the light band with lower effective mass is more conducive to carrier transport than the heavy band with higher effective mass, resulting in a significant increase in mobility. The comprehensive effect is that after La doping, the electrical conductivity increases up to 60 S/cm, and the Seebeck coefficient decreases obviously at room temperature but increases rapidly at high temperatures (600–923 K). The maximum power factor is ~0.41 mWm^−1^K^−2^, and the *ZT* value can reach 0.74 (923 K). Sb is also used to dope BiCuSeO as a congener of Bi in our previous work [16]. The band gap narrowed, as shown in Figure 4 and Figure 5, and the peaks of Cu/Se orbitals in the decomposed partial density of states were broadened after the Bi site was replaced by the element Sb. This resulted in an obvious increase in carrier concentration and electrical conductivity, as shown in Figure 6, while keeping a relatively high Seebeck coefficient and low thermal conductivity. The maximum power factor of 0.36 mWm^−1^K^−2^ and *ZT* value of 0.73 was reached for the Bi_0.95_Sb_0.05_CuSeO at 873 K, which was 65% higher than that of the undoped BiCuSeO ceramic. In addition, due to the positive valence of Cu in BiCuSeO, Ag doping at the Cu site also belongs to equivalent element doping, with a power factor of up to ~0.32 mWm^−1^K^−2^ and a *ZT* value of up to 0.64 (923 K). In doing so, similar to La doping, the *ZT* is increased to 0.6 at 800 K [51]. According to defect chemistry theory, when it comes to enhancing carrier concentration, equivalent element doping generally proves to be less effective than low-valence element doping. The underlying mechanism reveals that low-valence element doping can more readily introduce charge carriers into the crystal lattice, thereby creating a more conducive environment for charge transport. In contrast, equivalent element doping, due to its similar valence state, has a more limited impact on altering the electronic structure and generating additional carriers. As a consequence, the overall improvement in thermoelectric properties achieved through equivalent element doping typically pales in comparison to that brought about by low-valence element doping, as the latter exerts a more profound influence on optimizing the material’s electrical conductivity and Seebeck coefficient, two critical parameters that determine thermoelectric performance.

### 4.3. Chemical Defect Engineering of BiCuSeO

In the BiCuSeO compound, the formation of vacancy defects is closely related to its crystal structure and elemental valence states. According to defect chemistry theory, Bi and Cu exhibit +3 and +1 valences in the lattice, respectively. This charge discrepancy leads to the emergence of copper vacancies (Cu vacancies) in the lattice to maintain electrical neutrality [15].

(1) $$\begin{array}{l} BiCu_{1-x}SeO={\left(Bi_{2}O_{2}\right)}^{2}+(Cu_{2(1-x)}Se_{2}{)}^{2(1+x)+}\\ +2xV_{Cu}^{+}+2xh^{+} \end{array}$$

When Bi^3+^ occupies cationic sites in the lattice, Cu^+^ vacancies (i.e., the absence of Cu^+^ ions) form spontaneously to compensate for the excess positive charge, with each Bi^3+^ introducing one Cu vacancy. This intrinsic defect represents a thermodynamically stable state to maintain crystal electrical neutrality. Regardless of the preparation method, high temperatures or rapid cooling processes exacerbate uneven atomic diffusion, leading to the formation of more Cu vacancies. For instance, high-temperature sintering in solid-state synthesis promotes the volatilization of Cu atoms, directly increasing the concentration of Cu vacancies; rapid quenching may freeze out-of-equilibrium defects, further increasing vacancy density. Studies by Liu and his team have demonstrated that actively controlling the Cu content below the standard stoichiometric ratio in the BiCuSeO system can significantly increase the Cu vacancy concentration in the lattice, thereby optimizing thermoelectric conversion efficiency. When the Cu content is lower than the theoretical value, the lattice lacks sufficient Cu^+^ ions to occupy cationic sites. To maintain electrical neutrality, the system spontaneously generates more intrinsic Cu vacancies. The *ZT* value rises from 0.5 (standard ratio) to 0.81 (923 K). This tuning strategy avoids disorder-induced damage to the lattice caused by traditional doping, maintaining high crystalline quality [12]. The mechanism of Bi vacancies is similar to that of Cu vacancies [52]. Mamoru et al. also realize Bi vacancies by controlling the amount of Bi raw materials [53]. As donor defects, Bi vacancies release electrons into the lattice, but in the p-type conduction mechanism of BiCuSeO. Their more significant role is to reduce the excessive formation of Cu vacancies through charge compensation. When the Bi content is lower than the theoretical value, the lattice lacks sufficient Bi^3+^ ions to occupy cationic sites. By balancing carrier concentration and transport capacity, *ZT* increased to 0.60 (773 K). Studies by Ishizawa et al. and their teams have shown that controlling the Se content below the standard stoichiometric ratio in the BiCuSeO system significantly increases the Se vacancy concentration in the lattice, but the impact on thermoelectric conversion efficiency is dualistic [53]. When Se is insufficient, the absence of anionic sites in the lattice leads to the formation of anion vacancies. To maintain electrical neutrality, Se vacancies act as double-donor defects, potentially compensating for a charge. The highest *ZT* was less than 0.2. This indicates that Se vacancy engineering struggles to optimize key performance parameters of p-type materials simultaneously, exhibiting a significant trade-off effect.

### 4.4. Dual Doping/Vacancies for BiCuSeO

The pursuit of high thermoelectric performance in BiCuSeO has driven the development of dual-doping strategies, which leverage synergistic effects between different dopants to optimize both electrical transport properties and thermal conductivity. By combining elements with distinct ionic radii, valences, and electronic structures, dual doping can simultaneously enhance carrier concentration, adjust band characteristics, and manipulate phonon scattering. This section explores the mechanistic foundations and performance outcomes of various dual-doping approaches in BiCuSeO, highlighting how multi-elemental modifications address the trade-offs inherent in single-dopant systems. Ca/Pb dual-doped BiCuSeO has the best thermoelectric performance [15]. On the one hand, Ca/Pb exhibits positive bivalence, which can increase the hole concentration substituting the Bi site. To enhance the power factor, Pb doping could maintain a high Seebeck coefficient due to increased effective mass, while enhancing the electrical conductivity more effectively than other dopants due to the delocalized 6s orbitals from the lone pair electrons in Pb. In addition, by reducing the lattice thermal conductivity, Ca is more effective due to both mass differences (mass fluctuations) and differences in size and interatomic coupling forces (strain field fluctuations) between the Ca atom and Bi. The maximum *ZT* (873 K) value is as high as 1.5, and the power factor basically reaches 1 mWm^−1^K^−2^ in the whole temperature range. Sun et al. doped BiCuSeO with Zn substitute at the Bi site and S substitute at the O site [54]. Zn^2+^ substituting Bi^3+^ could enhance the hole concentration for non-isoelectronic replacement, while S substituting O could yield a large Seebeck coefficient. The maximum power factor is increased to 0.46 mWm^−1^K^−2^ and the corresponding maximum *ZT* is 0.68 (750 K). Wen et al. adopted a Ba substitute at the Bi site and a Ni substitute at the Cu site, as Ba^2+^ substituting Bi^3+^ as acceptors could increase the hole concentration, while magnetic Ni^+^ could give rise to extra spin entropy and the corresponding Seebeck coefficient [55]. The maximum power factor is increased to 0.57 mWm^−1^K^−2^ and the corresponding maximum *ZT* is 0.98 (923 K). Li/Mn dual doping is similar to Ba/Ni doping with the highest *ZT* of 0.9 at 873 K [56].

We used Ba/Pb to dope at the Bi site to increase the thermoelectric properties of BiCuSeO [57]. Barium (Ba) has proven to be an efficient dopant in thermoelectric materials. Its lower valence state contributes to the enhancement of electrical transport properties by modifying the electronic structure, while its relatively large atomic mass plays a crucial role in reducing thermal conductivity through increased phonon scattering. This dual effect of Ba doping makes it a valuable additive for optimizing the thermoelectric figure of merit. Regarding lead (Pb) doping, it is particularly notable for its ability to preserve a high Seebeck coefficient. This is attributed to the increase in the effective mass of charge carriers, which is beneficial for maintaining a significant thermoelectric voltage generation. Moreover, compared to many other dopants, Pb doping demonstrates a more pronounced enhancement in electrical conductivity. The origin of this superior conductivity improvement lies in the delocalized 6s orbitals derived from the lone pair of electrons of Pb. These delocalized orbitals facilitate the easier movement of charge carriers, thereby boosting the overall electrical conductivity of the doped materials. The maximum power factor is increased to 0.66 mWm^−1^K^−2^ and the corresponding maximum *ZT* is 1.01 (873 K). In addition, Te doping at the Se site can profoundly reduce the difference in electronegativity in the (Cu_2_Se_2_)^2-^ layer, increase bond covalency, and then increase the electrical conductivity. We adopted Ba/Te dual doping BiCuSeO [58]. The maximum power factor is increased to 0.72 mWm^−1^K^−2^ and the corresponding maximum *ZT* is 1.07 (873 K). And for that, Sb/Te doping can both tune the Fermi level and promote band convergence, decreasing the band gap and concurrently enhancing the carrier concentration; we used Sb/Te dual doping for BiCuSeO [17]. The maximum power factor is increased to 0.69 mWm^−1^K^−2^ and the corresponding maximum *ZT* is 1.04 (873 K).

We used Bi vacancies as acceptors (aimed at increasing hole concentration) combined with Fe doped at the Cu site (aimed at increasing the Seebeck coefficient) [59]. The maximum power factor is increased to 0.38 mWm^−1^K^−2^ and the corresponding maximum *ZT* is 0.76 (873 K). Li et al. adopted Bi/Cu dual vacancies [60]. On the one hand, dual vacancies as acceptors can increase the hole concentration; dual vacancies can enhance phonon scattering and reduce thermal conductivity. It is worth mentioning that the clearcut evidence in positron annihilation unambiguously confirms the interlayer charge transfer between these Bi/Cu dual vacancies, which results in a significant increase in electrical conductivity with a relatively high Seebeck coefficient. The maximum *ZT* is increased to 0.84 (873 K). Yb doping contains Yb^2+^ and Yb^3+^; the *ZT* is increased to 0.62 at 823 K using the variable valence element doping strategy [45]. F. Li et al. adopted Pb/La dual doping with Pb doping aimed at improving carrier concentration and La doping aimed at improving mobility [61]. The ZT has been increased to 0.9 at 873 K.

### 4.5. The Research Status of N-Type BiCuSeO

N-type BiCuSeO has not been developed successfully for a long time. According to defect chemistry theory, doping the Bi site with elements with a valence greater than +3, doping the Cu site with elements with a valence greater than +1, or doping the Se/O sites with negative monovalence could introduce electrons to realize N-type conversion of BiCuSeO. Novitskii et al. [21] used NaF as raw material for Na doping in BiCuSeO and unexpectedly found the N-type doping effect of F ions. Zhou et al. used BiClO as raw material to introduce Cl ions in order to introduce electrons [62], but the electron concentration introduced was not high enough to achieve N-type conversion. Zhao et al., by adopting halogen doping, have lowered the Seebeck coefficient of BiCuSeO to −500 µVK^−1^. However, the samples are not stable for the weak bonding for Cu- halogens [63]. Zhang et al. introduce additional Bi and Cu into BiCuSeO to fill the Bi/Cu vacancies (acceptors) in its structure in order to reduce the hole concentration and introduce electrons into BiCuSeO using Br/I doping at the Se site [64]. In this way, N-type BiCuSeO was successfully prepared by this three-position substitution method. The maximum Seebeck coefficient is −543 µVK^−1^ for Bi_1.04_Cu_1.05_Se_0.99_Br_0.01_O. However, this method only realizes N-type conversion in the temperature range of ~350–750 K, and the thermoelectric performance is very low, with a maximum *ZT* value of 0.07 (475 K) after compounding with Ag. Pan et al. realized N-type conversion by introducing electrons by Fe doping at the Cu site [65]. The maximum Seebeck coefficient was −53 µVK^−1^. However, the range of N-type conversion is still narrow (~350–500 K). In summary, the research on N-type BiCuSeO is not perfect. On the one hand, the temperature range for N-type conversion is very narrow. The corresponding thermoelectric performance is very low. Multi-position coordinated adjustment should be an efficient approach.

### 4.6. Structural Adjustment for BiCuSeO

The optimization of thermoelectric properties in BiCuSeO extends beyond elemental doping to encompass microstructural engineering strategies, which manipulate grain size, orientation, and composite architecture to synergistically enhance carrier transport and phonon scattering. These approaches address the intrinsic trade-offs between electrical conductivity, Seebeck coefficient, and thermal conductivity by leveraging nanostructuring, texturing, and modulation doping. This section explores the mechanistic basis and performance gains of these microstructural engineering techniques in BiCuSeO, highlighting their unique contributions to overcoming material limitations. In previous studies of thermoelectric materials, grain refinement was generally based on the idea of nanostructure engineering to enhance phonon scattering and reduce thermal conductivity [66]. For BiCuSeO, grain refinement has another advantage; with grain refinement, Cu vacancies (as acceptors) will increase, which would lead to an increase in hole concentration. In our study, the power factor of undoped samples increases from 0.26 mWm^−1^K^−2^ to 0.31 mWm^−1^K^−2^ and *ZT* from 0.43 to 0.62 by grain refinement (average grain size decreases from 1481 nm to 400 nm). In another of our studies, the power factor of Sb/Te-doped samples increases from 0.69 mWm^−1^K^−2^ to 0.72 mWm^−1^K^−2^ and *ZT* from 1.04 to 1.19 by grain refinement (average grain size decreases from 1445 nm to 387 nm) [20].

**Figure 7:**
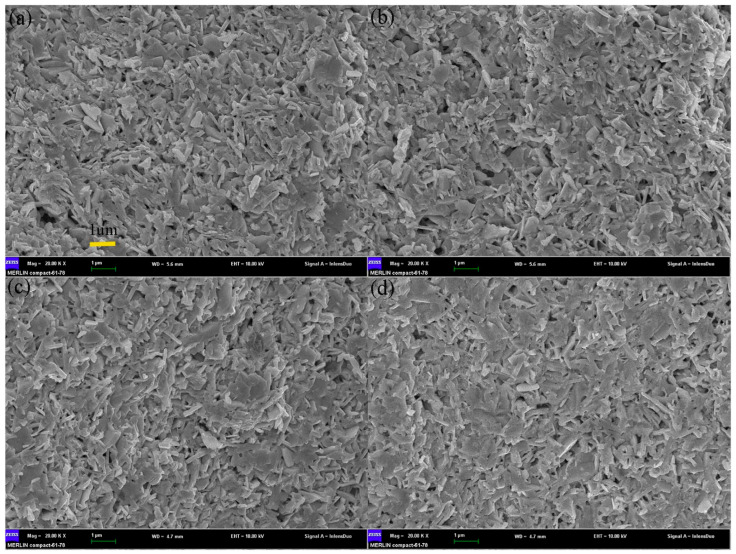
SEM of the fractured surfaces for BCSO bulks ((**a**–**d**) stand for the pristine sample, the uniformly doped sample, the heavily doped sample, and the modulation doped sample, respectively) [58].

**Figure 8:**
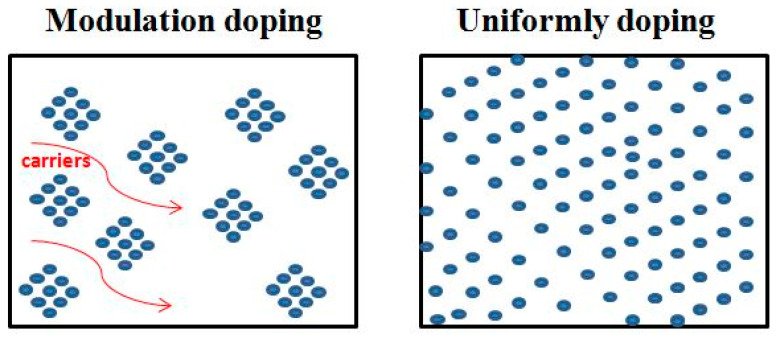
Schematic showing for the carrier transport of the uniformly doped sample and the modulation doped sample [58].

**Figure 9:**
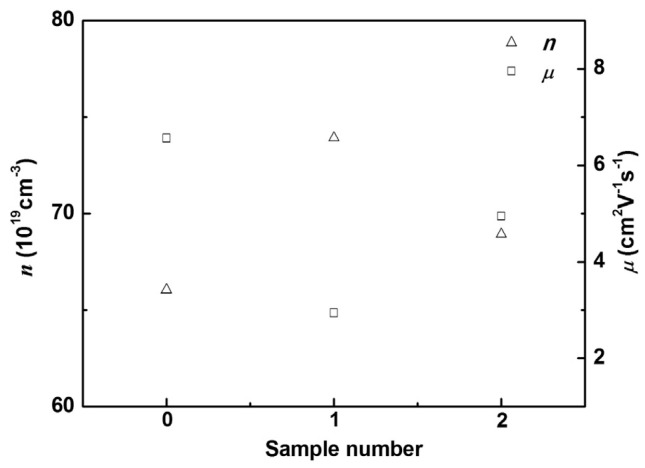
The carrier concentration (n), mobility (μ) for the uniformly doped sample, the heavily doped sample, and the modulation doped samplesat room temperature [58].

Three-dimensional modulation doping is an important method to improve carrier mobility. Carrier concentration and mobility are negatively correlated. Undoped samples generally have low carrier concentration and high mobility, while heavily doped samples have high carrier concentration and low mobility. The SEM of modulated doping samples is shown in Figure 7. The idea of modulation doping is to mix the undoped and heavily doped samples to obtain the modulation-doped samples, which have a relatively high carrier concentration and relatively high mobility (because carriers tend to migrate to regions with higher mobility, as shown in Figure 8). The group adopted modulation doping to improve the highest power factor of Ba/Te-doped samples from 0.72 mWm^−1^K^−2^ to 0.98 mWm^−1^K^−2^ and *ZT* from 1.07 to 1.17 [58]. When compared to the uniformly doped Bi₀.₉₀Ba_0 10_CuSe_0 90_Te_0 10_O, the heavily doped Bi_0 80_Ba_0 20_CuSe_0 80_Te_0 20_O shows a decrease in the Seebeck coefficient to 92 μV·K^−1^ near room temperature and 171 μV·K^−1^ at 873 K, which is attributed to the increase in carrier concentration, as shown in Figure 9. Although the carrier concentration (n) of the modulation-doped Bi_0 90_Ba_0 10_CuSe_0 90_Te_0 10_O is higher than that of the uniformly doped Bi_0 90_Ba_0 10_CuSe_0 90_Te_0 10_O, its Seebeck coefficient is actually higher, ranging from 111 μV·K⁻¹ near room temperature to 196 μV·K^−1^ at 873 K. The modulation method does enhance carrier mobility by twice without reducing carrier concentration. Thanks to the improved electrical conductivity and the almost unchanged Seebeck coefficient, a wide range and high power factor of 5–10 μW·cm^−1^·K^−2^ are achieved. Combined with the low thermal conductivity of approximately 0.25 W·m^−1^·K^−1^, this finally enables the BiCuSeO system to exhibit a high ZT value of about 1.4 at 923 K [67].

**Figure 10:**
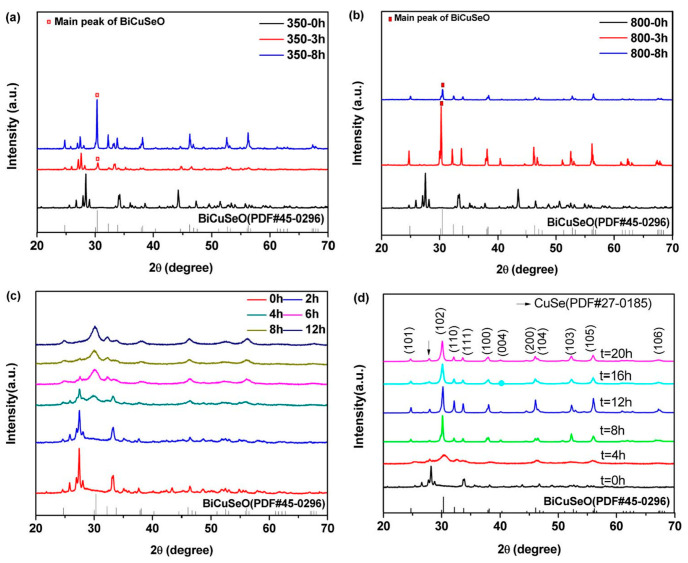
RD patterns from (**a**) annealing at 623 K, (**b**) annealing at 1023 K, (**c**) balling milling at 300 rpm, (**d**) balling milling at 400 rpm [69].

**Figure 11:**
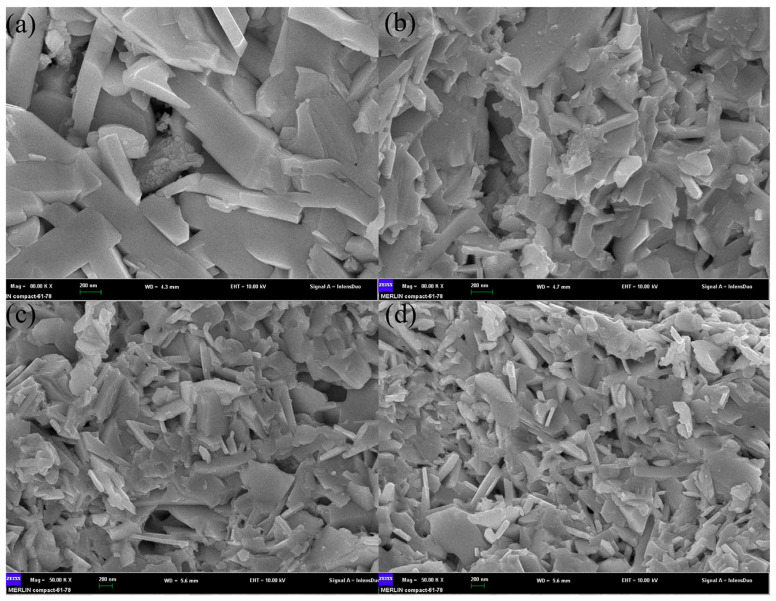
SEM of the fractured surfaces for BCSO bulks ((**a**) stands for 800 °C annealing; (**b**–**d**) stand for ball milling time t = 8 h, 12 h, 16 h, respectively) [69].

**Figure 12:**
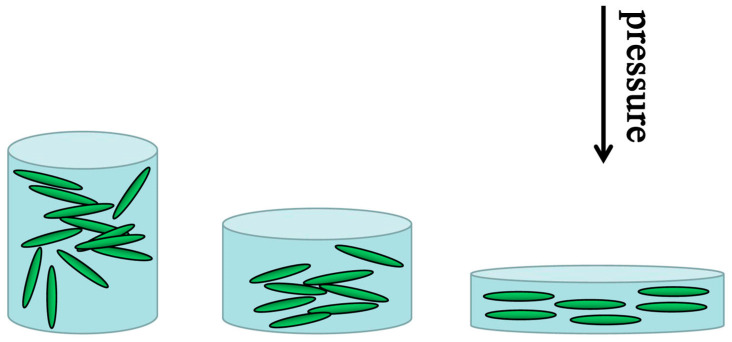
Schematic showing the texturing process.

Texturation (increasing mobility by reducing carrier scattering by promoting directional grain alignment) is a common method to improve the properties of thermoelectric materials [68]. As a kind of ceramic material, BiCuSeO has low plasticity and is not very convenient for promoting the orientation of grains. Zhao et al. promoted the arrangement of BiCuSeO grains by hot forging them three times [14]. Feng et al. promoting optimal grain orientation and texture formation through mechanical alloying (the crystal orientation XRD and SEM images are as shown in Figure 10 and Figure 11) are used to assist in detecting changes in the preferred orientation of BiCuSeO grains [69] are used to assist in detecting changes in the preferred orientation of BiCuSeO grains. In XRD analysis, the relative intensity of diffraction peaks reflects the degree of crystallographic orientation. When grains exhibit preferred orientation, certain crystal planes (e.g., (hkl) planes) tend to align in specific directions, causing significant increases or decreases in the corresponding peak intensities compared to standard powder diffraction data. EBSD, as a high-resolution microstructure characterization technique, directly measures the crystallographic orientation of individual grains and constructs pole figures to visually represent the distribution and concentration of crystal orientations. The overlap and density of pole figure arcs or spots quantify the strength of the preferred orientation: concentrated arcs indicate strong orientation, while scattered distributions suggest random orientation. Combined, these two methods provide complementary insights—XRD offers statistical orientation information for the entire sample, while EBSD enables microscale analysis of orientation heterogeneity—thereby comprehensively characterizing the evolution of grain-preferred orientation in BiCuSeO materials. The disk sample is hot-pressed into a larger-diameter disk sample in a larger-diameter die at high temperatures (as shown in Figure 12). In this way, the highest power factor of Ba-doped samples was increased from 0.63 mWm^−1^K^−2^ to 0.81 mWm^−1^K^−2^ and *ZT* from 1.1 to 1.4 (923 K). However, because this method is difficult to operate, no other researchers used this method for further study.

The idea of the composites is to introduce a kind of well-conductive material (often nano-dispersed phase) into the matrix to enhance the conductivity and phonon scattering, thereby enhancing the thermoelectric properties [70]. Liu et al. introduced nano-inclusions of copper selenides in BiCuSeO in order to combine the high conductivity of Cu_2_Se with the high Seebeck coefficient of BiCuSeO [71]. In this way, the highest *ZT* increased from 0.33 to 0.44 (923 K). Farooq et al. composited BiCuSeO with La_0.98_Sr_0.02_CoO_3_ [72], but the effect was very unsatisfactory. The highest *ZT* reached was as low as 0.07.

## 5. Summary and Outlook

As a new type of oxide thermoelectric material, BiCuSeO has wide application prospects due to its low thermal conductivity and high Seebeck coefficient. However, the low electric conductivity of BiCuSeO has limited the improvement of its thermoelectric performance. At present, the research on BiCuSeO thermoelectric performance mainly focuses on enhancing the power factor, especially on the optimization of the electric conductivity. Researchers have effectively modified the properties in the aspects of preparation methods, element doping, vacancy introduction, bandgap adjustment, and structure optimization. From the perspective of future development trends, future research directions can be carried out around the following points:

(1) Adjust the energy band structure and improve the power factor while maintaining low thermal conductivity. We can learn from the optimization methods of the thermoelectric performance of PbTe and Mg_2_Si, such as the effective band convergence [73]. In addition, simulation software can be used to predict the energy band structure and thermoelectric performances before the experiment, increase the types of doping elements used, and improve the efficiency and scope of research.

(2) Optimize the synthesis method. The mechanical alloying method has proven to be a valuable technique for laboratory investigations and small-scale production. Nevertheless, it faces significant challenges when it comes to large-scale applications. One of the primary obstacles is the potential contamination introduced by the ball milling media, which can compromise the purity and properties of the synthesized materials. Self-propagating combustion synthesis emerges as a highly promising preparation route, distinguished by its rapid hot-pressing process, one-step forming capability, short reaction duration, and low energy consumption. Incorporating this method into the preparation of BiCuSeO materials in future research holds great potential for not only augmenting the thermoelectric performance of the materials but also reducing manufacturing costs. However, it should be noted that the current self-propagating technology tailored for BiCuSeO materials is still in a state of development and refinement. Notably, while the hot deformation process has been shown to improve both the grain orientation and carrier mobility of BiCuSeO compounds, the enhancement in mobility remains relatively limited due to the suboptimal degree of orientation. Additionally, the pronounced disparity in the degree of orientation across different regions of the material poses a formidable challenge in obtaining bulk materials with homogeneous properties, thereby impeding the practical implementation of this method. In the pursuit of advancing the field of BiCuSeO-based thermoelectric materials, the successful synthesis of single-crystal materials with superior thermoelectric properties would represent a significant breakthrough. Such single crystals would not only offer the potential for further optimizing thermoelectric performance but also facilitate the transition towards large-scale production, thereby bringing us closer to the realization of practical applications of BiCuSeO-based thermoelectric devices.

(3) Explore new materials with similar structures, such as BiCuTeO, BiCuSO, LaCuSeO, and CeCuSeO. BiCuTeO has demonstrated solid thermoelectric properties. Based on this, we can investigate multi-site doping, texturing, and modulation doping to further improve its thermoelectric properties. BiCuSO and LaCuSeO band gaps are too wide. Bi/La/S/Se sites can be partially replaced by other elements to reduce the band gap and optimize energy band structure and thermoelectric properties.

(4) Use multi-position doping to optimize the performance of N-type BiCuSeO. According to the current studies, the effect of single-position doping is very unsatisfactory. It is necessary to dope in two, three, or even four positions. According to defect chemistry theory, the Bi site can be doped with elements of valence greater than +3, the Cu site with a valence greater than +1, and the Se or O sites with negative monovalent elements to further optimize the N-type conversion and the corresponding thermoelectric properties.

(5) Perform the research of actual production of BiCuSeO. BiCuSeO devices have been applied in the field of thermoelectric catalysis [74], and the corresponding device research needs to be carried out. We should also study the related issues of thermal stability, electrode materials, contact resistance, and mechanical properties in the process of device preparation and application to promote the commercial application of BiCuSeO.

## Data Availability

The raw/processed data required to reproduce these findings cannot be shared at this time as the data also forms part of an ongoing study.
